# α-Conotoxin TxIB Reversed Nicotine-Induced Locomotor Sensitization and Nicotine-Enhanced Dopaminergic Activity in Mice

**DOI:** 10.3390/md23030109

**Published:** 2025-03-04

**Authors:** Weifeng Xu, Meiting Wang, Xiaodan Li, Rongyan He, Ren-Bo Ding, Jiaolin Bao, Dongting Zhangsun, Sulan Luo

**Affiliations:** 1 Key Laboratory of Tropical Biological Resources of Ministry of Education, Hainan University, Haikou 570228, China; 19071010110011@hainanu.edu.cn (W.X.); 993586@hainanu.edu.cn (X.L.); dingrenbo@hainanu.edu.cn (R.-B.D.); baojiaolin@hainanu.edu.cn (J.B.); 2 Guangxi Key Laboratory of Special Biomedicine, School of Medicine, Guangxi University, Nanning 530004, China; wmttymt@163.com (M.W.); herongyan@gxu.edu.cn (R.H.)

**Keywords:** α-CTx TxIB, α6β2* nAChRs, nicotine addiction, locomotor sensitization, dopamine

## Abstract

Nicotine addiction is a serious global public health problem, so there is an urgent necessity to develop novel effective smoking cessation treatments with fewer adverse effects. Spontaneous behavioral sensitization induced by repeated intermittent exposure to the addictive substance represents a classical animal model of addiction research. A significant contributor to nicotine addiction is its interaction with α6β2* nAChRs located on midbrain dopaminergic neurons, which leads to an increase in dopamine (DA) release. α-Conotoxin (α-CTx) TxIB is a novel potent antagonist of the α6/α3β2β3* nAChRs, with an IC50 value of 28.4 nM developed by our group. In this study, we aimed to investigate the effectiveness of α-CTx TxIB in countering nicotine-induced behavioral sensitization and moderating the impact of nicotine on dopamine accumulation in the midbrain. Our results demonstrated that repeated nicotine administration remarkably elevated the locomotor activity of mice, including the number of entries, average speed, and total distance traveled, which could be effectively attenuated by α-CTx TxIB intervention in a dose-dependent manner (1 nmol and 5 nmol TxIB per mouse). Furthermore, 5 nmol α-CTx TxIB significantly reduced the nicotine-elevated DA and norepinephrine (NE) levels in the ventral tegmental area (VTA) and nucleus accumbens (NAc) of mice. 5 nmol α-CTx TxIB also markedly decreased the expression of critical proteins such as the dopamine transporter (DAT), N-methyl-D-aspartic acid receptor (NMDAR), and c-Fos in the NAc and prefrontal cortex (PFC) of the nicotine-exposed mice. This research provided the first compelling evidence that α-CTx TxIB attenuated nicotine-induced locomotor sensitization and inhibited the nicotine-induced dopamine elevation in mice. These results open up new avenues for exploring the therapeutic potential of α-CTx TxIB in the treatment of nicotine addiction.

## 1. Introduction

Although the World Health Organization Framework Convention on Tobacco Control (FCTC) has been in place for more than twenty years, there are still around 1.2 billion smokers globally as reported by the Global Burden of Diseases, Injuries, and Risk Factors Study (GBD) 2019. The report also indicated that about 7 million and 1 million deaths each year are associated with smoking and secondhand smoke, respectively [1,2]. This enduring issue highlights the ongoing challenges in effectively reducing tobacco consumption, despite the long-standing efforts of international health initiatives. Tobacco product use is linked to a heightened risk of various diseases, with estimates suggesting that smokers may lose an average of no less than 10 years of life expectancy in comparison to the non-smoking population [3]. Conversely, quitting smoking significantly reduces the likelihood of developing smoking-related health issues and can lead to increased life expectancy. Individuals who stop smoking by the age of 50 can expect an additional 6 years of life compared to those who continue smoking [3,4]. Upon learning about the risks associated with tobacco, a significant proportion of smokers (over 70%) in America express a desire to quit smoking [5]. However, the process of quitting smoking naturally is often difficult and generally requires no less than ten years of sustained effort and at least ten attempts [5,6,7,8]. Nonetheless, with appropriate clinical support, 10–30% of smokers can successfully achieve long-term cessation [7]. Therefore, there is an urgent necessity to develop more effective smoking cessation treatments with fewer adverse effects.

Nicotine is the main psychoactive ingredient in tobacco. Nicotine rapidly binds to acetylcholine receptors (nAChRs) in the central nervous system and triggers an increase in dopamine (DA) release during smoking [9]. nAChRs consist of pentameric ion channels that are gated by ligands and primarily found in the central and the peripheral nervous systems [10,11]. An increasing number of studies have focused on α6β2*nAChRs because they are abundantly expressed on dopaminergic neurons, affecting dopamine release and fostering the rewarding effects of nicotine [12,13,14,15,16,17,18,19]. Previous studies indicate that α6β2* nAChRs play a critical role in regulating dopamine release within midbrain and mesostriatal terminals [13,20,21,22]. Additionally, it has been discovered that inhibiting α6β2*nAChRs reduced nicotine cravings in vivo in the conditioned place preference (CPP) and self-administration (SA) models [22,23,24].

TxIB, an α-conotoxin (α-CTx), was developed by our group from the genomic DNA of Conus textile. This peptide acts as a potent selective antagonist of the α6/α3β2β3* nAChRs, with an IC50 value of 28.4 nM [25]. A series of investigations were conducted on TxIB to examine its pharmacological effects in the elevated plus maze test, open field test, tail suspension test, and water maze test in vivo. The findings demonstrated that TxIB did not impede spontaneous movement, did not elicit anxiety or depressive behavior, and did not affect cognitive functions such as learning and memory [26]. Previous research has showed that TxIB could suppress the nicotine-induced expression of conditioned CPP in mice [27].

In the present study, a behavioral sensitization model was employed to examine the effects of TxIB in attenuating nicotine-induced locomotor sensitization and the associated elevation in dopaminergic activity in mice.

## 2. Results

### 2.1. Purification and Structural Identification of α-Conotoxin TxIB

**Figure 1:**
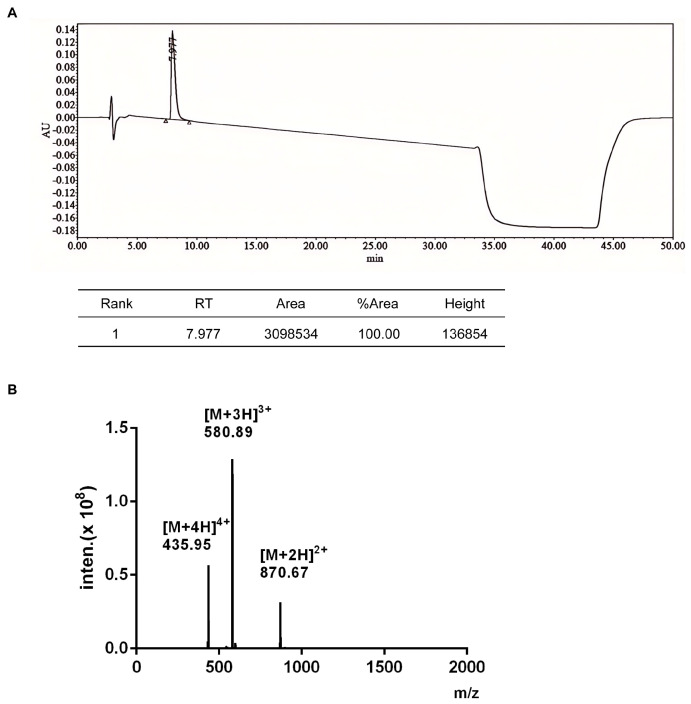
The HPLC profile and the ESI-MS spectrometry of α-conotoxin TxIB. Peptides were analyzed on a reversed-phase C18 Vydac column using a linear gradient of ACN: 0–30 min 5−40% buffer B (buffer B is 0.05% TFA in 90% ACN and 9.95% ddH_2_O). Buffer A is 0.075% TFA in ddH_2_O. (**A**) The HPLC traces for TxIB. (**B**) ESI-MS data of TxIB show the *m*/*z* value of [M + 3H]^3+^ is 580.89, which corresponds with the observed mass of 1739.67 Da (theoretical mass is 1739.70 Da).

α-Conotoxin TxIB was developed from Conus textile in Hainan through gene cloning by our group [25]. The α-CTx TxIB sequence is GCCSDPPCRNKHPDLC#, with # indicating the C-terminal carboxamide [25]. Following a two-step oxidation process, the linear peptide was purified to a purity exceeding 99%, meeting the detection criteria required for subsequent experiments. As illustrated in Figure 1A, the main peaks were collected via HPLC. The retention time of fully oxidized TxIB was 7.977 min, with a purity of 100%. As shown in Figure 1B, the relative molecular mass detected by electrospray ionization mass spectrometry (1739.67 Da) was compared with the theoretical relative molecular mass (1739.70 Da), and the error was less than 1 Da.

### 2.2. α-Conotoxin TxIB Attenuated Nicotine-Induced Locomotor Sensitization in Mice

**Figure 2:**
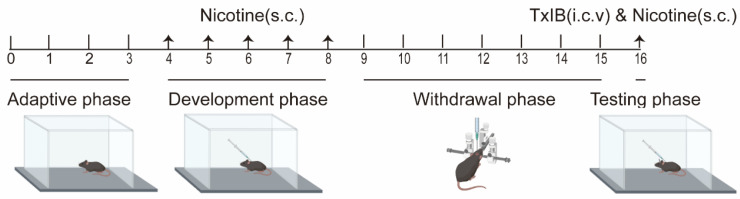
Schedule of nicotine-induced locomotor sensitization experiments. Mice were subcutaneously injected 0.5 mg/kg nicotine during the development phase and testing phase. During the test period, mice were administered TxIB 30 min before nicotine administration, and behavioral tests were performed immediately.

To assess the effect of α-CTx TxIB on nicotine-induced sensitization in mice, a model for locomotor sensitization was established. The experimental timeline included four stages: an adaptive stage lasting three days, a development stage of five days in which nicotine (0.5 mg/kg) was administered to the mice, a withdrawal stage lasting seven days, and a testing phase of one day (Figure 2).

**Figure 3:**
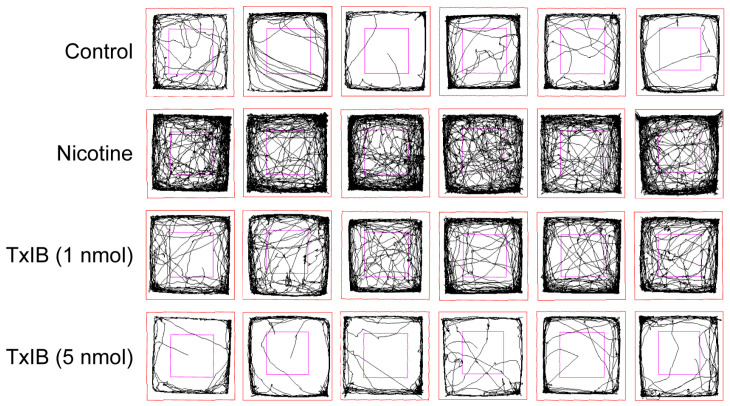
Two-dimensional motion track. Red outer boxes mark spontaneous activity boxes, purple inner boxes mark the central region of each box, and black lines mark mouse movement trajectories.

**Figure 4:**
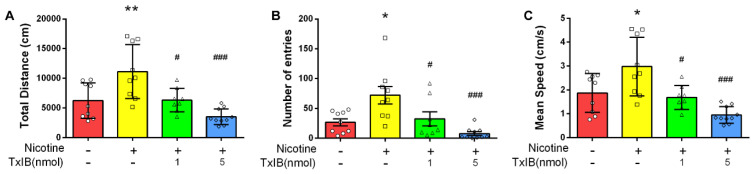
α-CTx TxIB attenuated nicotine-induced locomotor sensitization in mice. Data represent mean ± S.E.M. for 8 mice. The red columns represent the control group, and ○ represents the data for each individual mouse in this group. The yellow columns represent the nicotine-treated group, and □ represents the data for each individual mouse in this group. The green columns represent the low-dose administration group, and △ represents the data for each individual mouse in this group. The blue columns represent the high-dose administration group, and ◇ represents the data for each individual mouse in this group. (**A**) The total distances of locomotor activity test. (**B**) The numbers of entries of locomotor activity test. (**C**) The mean speeds of locomotor activity test. * denotes a significant difference from the Ctrl group; # denotes a significant difference from the nicotine-treated group (* *p* < 0.05, ** *p* < 0.01, # *p* < 0.05, ### *p* < 0.001).

The 2D traces illustrated the elevated locomotor activity in the nicotine-treated group, which was dose-dependently attenuated by TxIB during the testing phase (Figure 3). In order to ascertain the motor activity of mice, three representative indicators were selected: total distance, number of entries to the central area, and mean speed (Figure 4). The means of the total distance of the mice in the control group, nicotine-treated group, and low-dose (1 nmol) and high-dose (5 nmol) α-CTx TxIB group were 6239, 11,128, and 6332 and 3524 cm, respectively (Figure 4A). The average counts of entries into the central region were recorded as 26.78, 98.11, and 32.50 and 7.80, respectively (Figure 4B). The mean speed records were 1.872, 2.977, and 1.684 and 0.952 cm/s, respectively (Figure 4C). These results indicated that the nicotine-treated group exhibited significant increases in total distance traveled, frequency of entries into the central area, and mean speed compared to the control group (*p* < 0.05). In contrast, the groups receiving low-dose (1 nmol) and high-dose (5 nmol) α-CTx TxIB demonstrated substantial reductions in these key measures compared to the nicotine-treated group, with *p*-values indicating significance (*p* < 0.05 for low dose and *p* < 0.001 for high dose). This reduction suggested that TxIB had a potent inhibitory effect on the heightened locomotor activity induced by nicotine. Overall, our results demonstrated that repeated nicotine administration significantly elevated the locomotor activity of mice, which could be effectively attenuated by TxIB intervention in a dose-dependent manner (Figure 3 and Figure 4).

### 2.3. α-Conotoxin TxIB Suppressed the Increase in DA and Norepinephrine (NE) in the Ventral Tegmental Area (VTA) and Nucleus Accumbens (NAc) of Mice Induced by Nicotine

**Figure 5:**
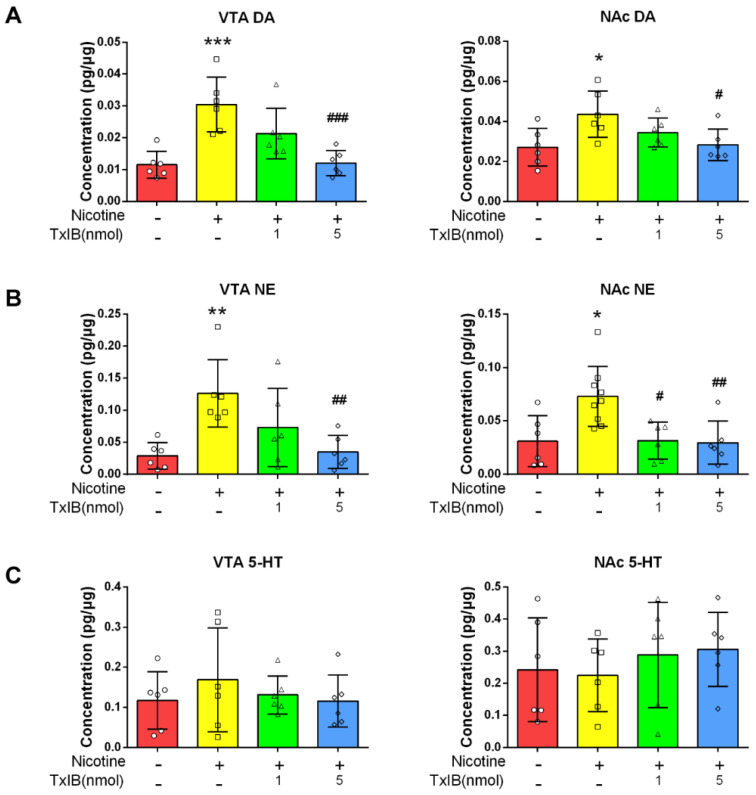
α-CTx TxIB suppressed the nicotine-induced increase of DA and NE levels in the VTA and NAc of mice. Data represent mean ± S.E.M. for 6 mice. The red columns represent the control group, and ○ represents the data for each individual mouse in this group. The yellow columns represent the nicotine-treated group, and □ represents the data for each individual mouse in this group. The green columns represent the low-dose administration group, and △ represents the data for each individual mouse in this group. The blue columns represent the high-dose administration group, and ◇ represents the data for each individual mouse in this group. (**A**) The DA concentrations in the VTA and NAc; (**B**) The NE concentrations in the VTA and NAc; (**C**) The 5-HT concentrations in the VTA and NAc. * denotes a significant difference from the control group; # denotes a significant difference from the nicotine-treated group (* *p* < 0.05, ** *p* < 0.01, *** *p* < 0.001, # *p* < 0.05, ## *p* < 0.01, ### *p* < 0.001).

Following behavioral assessments, brain regions were dissected to measure neurotransmitter changes. DA levels in the ventral tegmental area (VTA)/nucleus accumbens (NAc) were quantified via ELISA. The dopamine content in the VTA and NAc of mice in the nicotine-treated group (0.03042 pg/μg and 0.04366 pg/μg, respectively) was significantly higher (*p* < 0.001 and *p* < 0.05, respectively) than that in the control group (0.01151 pg/μg and 0.02714 pg/μg, respectively), and 5 nmol α-CTx TxIB-treated group (0.01203 pg/μg and 0.02840 pg/μg; *p* < 0.001 and *p* < 0.05, respectively) and were observed to prevent the accumulation of DA in these brain regions (Figure 5A).

It has been demonstrated that excessive accumulation of DA results in the synthesis of norepinephrine (NE) [28]. Therefore, we further investigated the content of NE in these brain regions. The results were similar to those of DA. The NE levels in the ventral VTA and NAc of nicotine-treated group (0.1262 pg/μg and 0.07293 pg/μg, respectively) were significantly higher (*p* < 0.01 and *p* < 0.05, respectively) compared to those in the control group (0.02902 pg/μg and 0.03099 pg/μg, respectively). Additionally, the 5 nmol α-CTx TxIB-treated group (0.03488 pg/μg and 0.02960 pg/μg; *p* < 0.01 and *p* < 0.01, respectively) was found to inhibit NE accumulation in these brain regions (Figure 5B). These data suggested that α-CTx TxIB had the potential to counteract the pronounced increase in NE concentration associated with nicotine exposure.

Given that serotonin (5-HT) accumulation has been demonstrated to inhibit locomotor activity [29], we subsequently examined the 5-HT contents in these brain regions. As illustrated in Figure 5C, no notable discrepancies were observed among the control, nicotine-treated, and TxIB groups.

Taken together, our results demonstrated that repeated intermittent administration of nicotine led to elevated levels of DA and NE in the VTA and NAc of mice. These increases could be effectively inhibited by α-CTx TxIB treatment. Additionally, neither nicotine nor TxIB had a significant effect on 5-HT accumulation.

### 2.4. α-Conotoxin TxIB Suppressed the Expression of Dopamine Transporter (DAT)/N-Methyl-D-Aspartate Receptor (NMDAR) and c-Fos in the NAc and Prefrontal Cortex (PFC) of Mice Induced by Nicotine

**Figure 6:**
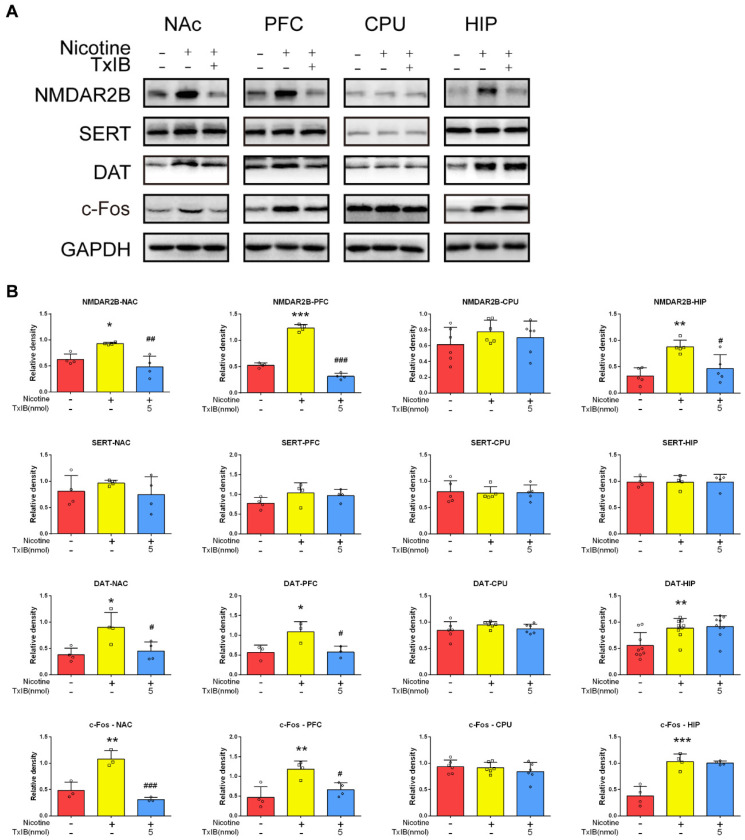
α-CTx TxIB suppressed the expressions of NMDAR2B/DAT and c-Fos in the VTA and NAc of mice induced by nicotine. Data represent mean ± S.E.M. for 3–9 mice. The red columns represent the control group, and ○ represents the data for each individual mouse in this group. The yellow columns represent the nicotine-treated group, and □ represents the data for each individual mouse in this group. The blue columns represent the 5 nmol TxIB administration group, and ◇ represents the data for each individual mouse in this group. (**A**) The expressions of NMDAR/SERT/DAT/c-Fos/GAPDH in NAc/PFC/CPU/HIP analyzed by Western blotting; (**B**) The densities relative to GAPDH of NMDAR/DAT and c-Fos in NAc/PFC/CPU/HIP. * denotes a significant difference from the control group; # denotes a significant difference from the nicotine-treated group (* *p* < 0.05, ** *p* < 0.01, *** *p* < 0.001, # *p* < 0.05, ## *p* < 0.01, ### *p* < 0.001).

Given that transmitter content is controlled by both secretion and transport mechanisms, the expression of DA/5-HT transporter proteins was assessed using Western blot analysis. Addictive behaviors are closely linked to the regulation of behavior and memory, with the prefrontal cortex (PFC) [30,31] and hippocampus (HIP) [32] playing crucial roles in these processes. The corpus striatum (CPU) is one of the brain regions in the central nervous system with relatively high densities of dopaminergic terminals and elevated DA concentrations [33]. The expression levels of the dopamine transporter (DAT) in the NAc and prefrontal cortex (PFC) of the nicotine-treated group (density relative to GAPDH: 0.9025 and 1.0870, respectively) were significantly higher (*p* < 0.05 for both) compared to that in the control group (density relative to GAPDH: 0.3800 and 0.5653, respectively). Additionally, the 5 nmol α-CTx TxIB-treated group (density relative to GAPDH: 0.4490 and 0.5781, respectively; *p* < 0.05 for both) was found to inhibit DAT accumulation in these brain regions (Figure 6). Moreover, the analysis revealed no significant differences in SERT expression across the control, nicotine-treated, and TxIB groups in these brain regions.

Glutamate serves as the principal excitatory neurotransmitter in the brain and is crucial for synaptic plasticity [34]. It has been shown that the activation of the N-methyl-D-aspartate receptor (NMDAR) influences dopamine (DA) projections [35]. Therefore, the expression of NMDARs in distinct brain regions was investigated. The expression of the NMDAR2B in the NAc, prefrontal PFC, and hippocampus (HIP) of the nicotine-treated group (density relative to GAPDH: 0.9306, 1.2330, and 0.8787, respectively) was significantly higher (*p* < 0.05, *p* < 0.0001, and *p* < 0.01, respectively) compared to that in the control group (density relative to GAPDH: 0.6278, 0.5255, and 0.3214, respectively). Additionally, the 5 nmol α-CTx TxIB-treated group (density relative to GAPDH: 0.4849, 0.3155, and 0.4616; *p* < 0.01, *p* < 0.0001, and *p* < 0.05, respectively) was found to inhibit the accumulation of NMDAR in these brain regions (Figure 6). The above results demonstrated that nicotine promoted NMDAR expression in the NAc/PFC/HIP, which could be reversed by TxIB intervention.

c-Fos is a protein that plays regulatory functions in neuronal cell growth and differentiation. In the neuroscience field, c-Fos is commonly used as a marker for neuronal activity [36]. Following neural stimulation, c-Fos levels increase rapidly, which is regarded as an essential signal of the nervous system responding to diverse stimuli [36]. Our study aimed to explore if TxIB counteracts the neuroadaptive changes brought by nicotine, as indicated by increased c-Fos expression. The expression of c-Fos in the NAc and PFC of the nicotine-treated group (density relative to GAPDH: 1.0800 and 1.1850, respectively) was significantly higher (*p* < 0.01 for both) compared to that in the control group (density relative to GAPDH: 0.4824 and 0.4679, respectively). Additionally, the 5 nmol α-CTx TxIB group (density relative to GAPDH: 0.3107 and 0.6645; *p* < 0.001 and *p* < 0.05, respectively) was found to inhibit c-Fos accumulation in these brain regions (Figure 6).

Taken together, our findings uncovered that repeated intermittent nicotine administration resulted in the elevated expression levels of NMDAR2B/DAT and c-Fos in the NAc and PFC regions of mice. This effect could be mitigated by α-CTx TxIB intervention at a dose of 5 nmol per mouse. Moreover, neither nicotine nor TxIB exerted a notable influence on serotonin accumulation.

## 3. Discussion

Decades of neurobiological research have established that nicotine exerts its primary effects via activation of brain nAChRs to modulate neurotransmission [37]. While α4β2* nAChRs are the predominant contributors to nicotine addiction, emerging evidence suggests a modulatory role of α6β2* nAChRs, in reinforcing addictive behaviors [38]. Electrophysiological evidence confirms β2 subunits drive nicotine-induced ventral tegmental area (VTA) neuronal activation [39,40]. Genetic deletion studies demonstrate α4 [39,41]/α6 [22,24] and β2 [39,42] subunits critically regulate nicotine-seeking behaviors. Utilizing α-conotoxin TxIB to block α6β2* nAChRs and attenuate nicotine-induced behavioral sensitization in mice, this study provided novel insights into the anti-addictive potential of targeting this receptor subtype.

The integration of multiple nicotine addiction-related behavioral models, including self-administration [43], conditioned place preference [44], withdrawal syndrome [45], and locomotor sensitization [46] in animals, allows for comprehensive elucidation of pharmacological and behavioral effects across the addiction cycle (craving, drug use, withdrawal, and relapse) [47], circumventing mechanistic biases inherent to single-model approaches [48], enriching neural circuitry dissection [39], and ultimately enhancing translational validity for clinical applications [49]. Due to its capacity to recapitulate neuroadaptive hallmarks of addictive drugs [50,51] and its high predictive validity in preclinical screening paradigms [52], behavioral sensitization has emerged as a critical preclinical model for evaluating anti-addiction therapeutic potential [53]. Building upon our prior investigation of TxIB’s efficacy in attenuating nicotine-induced CPP in mice, the present study extended this line of inquiry by evaluating the properties of TxIB against chronic nicotine-induced behavioral sensitization.

As Table 1 illustrates, TxIB attenuated the expression of nicotine-induced locomotor sensitization without affecting spontaneous movement, anxiety, depression, or cognitive function. In contrast to previously reported compounds demonstrating efficacy against nicotine-induced behavioral sensitization, including salmon calcitonin [54], α-conotoxin AuIB (which blocks α3β4* nAChRs) [55], and the D1R antagonist SCH-2339040 [56], TxIB introduced a novel molecular target (α6β2* nAChR subtype), thereby expanding the target repertoire for nicotine. However, whether TxIB can attenuate the acquisition of nicotine-induced behavioral sensitization and counteract acute nicotine-evoked locomotor hyperactivity remains to be systematically investigated.

The neurobiological mechanisms underlying nicotine-induced behavioral sensitization have been extensively studied, with the β2-nAChR-dependent activation of dopaminergic neurons in the VTA, enhanced dopamine release, and subsequent dopaminergic projections to the NAc being well-established components of this process [39,57,58]. Distinct from established methodologies such as brain slice electrophysiology [39] and HPLC with electrochemical detection [59], this study utilized ELISA-based quantification of DA concentrations in the VTA and NAc after behavioral tests in mice. In concert with prior findings of enhanced DA release [59], our data demonstrated that repeated intermittent nicotine administration induces DA elevation in both the VTA and NAc. DAT upregulation acts as a compensatory mechanism to counteract chronic DA hyperexocytosis, thereby normalizing synaptic DA dynamics [60,61]. In this study, the observed DA concentrations in the NAc of mice exhibited coordinated changes with DAT levels, indicating that TxIB effectively counteracted the dopaminergic activity increase induced by repeated intermittent nicotine administration. Notably, Perez et al. previously reported that long-term nicotine exposure reduces α6β2* nAChR-mediated dopamine release in the NAc of nonhuman primates [40]. Further exploration of TxIB’s anti-nicotine addiction efficacy and mechanisms—including validation in alternative animal models and investigation—remains imperative.

**Figure 7:**
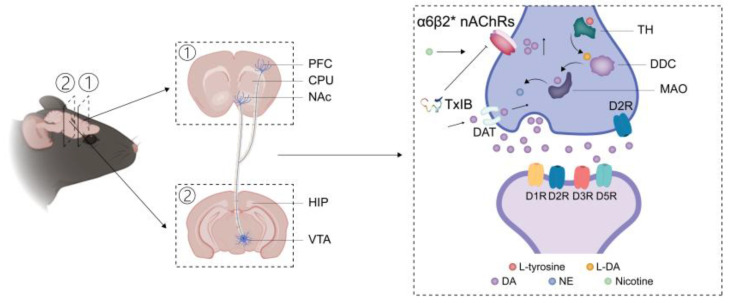
The proposed mechanism of α-conotoxin TxIB in reversing the expression of nicotine-induced behavioral sensitization. Nicotine activates α6β2* nAChRs, inducing the release of both DA and NE. α-Conotoxin TxIB antagonizes α6β2* nAChRs, reversing nicotine-induced increases in dopaminergic activity.

Although the activation of VTA dopaminergic neurons, which enhances locus coeruleus (LC) noradrenergic neuron firing via prefrontal cortex-LC circuitry, is a recognized mechanism underlying DA-NE system synergy in reward and stress processing [62], the biochemical pathway from tyrosine hydroxylase (TH) to DA synthesis has been underexplored in nicotine addiction research. The synthesis of dopamine from L-tyrosine is a well-established biochemical pathway that involves two critical enzymatic steps. At the outset, the enzyme TH facilitates the conversion of L-tyrosine into L-DOPA (L-3,4-dihydroxyphenylalanine), a process regarded as the rate-limiting stage in the production of catecholamines [63]. Subsequently, dopamine is produced through the decarboxylation of L-DOPA by the enzyme Dopa decarboxylase (DDC) [64]. Under the catalysis of dopamine β-hydroxylase (DβH), DA generates NE, which also promotes behavioral sensitization [28]. As illustrated in Figure 7, a potential mechanism of TxIB in reversing the expression of nicotine-induced behavioral sensitization has been proposed. However, further experimental validation—such as quantifying the expression levels of DβH and TH and monoamine oxidase (MAO)—would be required to confirm this hypothesis. The reliance on ELISA rather than direct measurement of DβH constitutes a study limitation. Nevertheless, existing evidence supports NE presence in the VTA: Wojciech et al. [65] identified DβH expression in the rat VTA, while Isingrini et al. [66] quantified NE levels in the mouse VTA using HPLC. These findings strengthen the validity of our approach.

The connection between NMDARs and behavioral sensitization represents a complicated interaction that has attracted considerable interest in neuroscience [35,67,68]. Consistent with cocaine- and amphetamine-induced behavioral sensitization—both of which upregulate NMDAR levels in the NAc [69,70]—this study demonstrated that nicotine comparably elevated NAc NMDAR expression during the development of behavioral sensitization in mice. It is noteworthy that the TxIB-treated group exhibited lower NMDAR levels compared to saline-treated controls. Whether TxIB directly interacts with NMDARs to mediate this downregulation requires further experimental validation through approaches such as electrophysiological recordings assessing effects of TxIB on NMDAR-evoked currents.

Consistent with the findings of Kiba and Jayaraman regarding the pattern of nicotine-induced c-Fos expression in the rat striatum and its relationship with DA receptors and NMDARs [71], this study additionally confirmed that in a behavioral sensitization model, nicotine increased c-Fos levels in the NAc/VTA and HIP. This study demonstrated that α-conotoxin TxIB inhibited nicotine-induced c-Fos upregulation, which provided a novel perspective for interpreting α6β2* nAChRs as a potential anti-nicotine addiction target.

Nicotine addiction mechanisms and the molecular pharmacology of TxIB represent complex scientific challenges. Our upcoming study will investigate the anti-addictive mechanism of TxIB by integrating multi-omics, electrophysiology, and radioimaging.

In summary, this study demonstrated that TxIB reversed repeated intermittent nicotine injection-induced behavioral sensitization and dopaminergic activity increase in mice, alongside attenuating nicotine-induced increases in NMDAR and c-Fos levels within the NAc. At the same time, these findings expanded the mechanistic framework of nicotine-induced behavioral sensitization.

## 4. Materials and Methods

### 4.1. Chemical Synthesis of α-Conotoxin TxIB

As previously described [25,27], the linear peptide TxIB underwent a two-step oxidative protocol employing potassium ferricyanide and iodine in sequential reactions to facilitate the formation of two distinct disulfide bridges. Following cyclization, the reaction mixture was purified through reversed-phase high-performance liquid chromatography (RP-HPLC) under gradient elution conditions. Structural validation of the bicyclic peptide was subsequently achieved by electrospray ionization mass spectrometry (ESI-MS), which confirmed both molecular integrity and oxidation state.

### 4.2. Animals

Male C57BL/6J mice (18–20 g, 6–8 weeks old) were obtained from Hunan SJA Laboratory Animal Co., Ltd., Changsha, China (approval no. SCXK 2016-0002). The mice were randomly grouped and housed in cages within a specific pathogen-free (SPF) facility for health and well-being.

Facility conditions were regulated: the lighting cycle was 12 h (8:00 AM–8:00 PM), the temperature was 23 ± 1 °C, and the humidity was 60–80%. Precise control is crucial for study reliability and mouse welfare.

Before the experiments, the mice were acclimatized for at least one week. All procedures adhered to Guangxi University IACUC standards (Approval No. GXU-2022-238; Approval date 28 March 2022), minimizing distress and using the fewest animals needed.

In this study, male C57BL/6 mice were stratified into four experimental cohorts: (1) saline control, (2) nicotine-treated group, (3) low-dose TxIB (1 nmol)-treated group, and (4) high-dose TxIB (5 nmol)-treated group, to evaluate the therapeutic potential of α-CTx TxIB against nicotine-induced behavioral and neurochemical alterations.

### 4.3. Nicotine-Induced Locomotor Sensitization

The experimental process of behavioral sensitization included four stages: the adaptive phase, development phase, withdrawal phase, and the testing phase [72].

Adaptive phase (Day 1–3): Following daily subcutaneous administration of normal saline (10 mL/kg), experimental mice were placed in the center of a standardized behavioral arena (30 × 40 × 40 cm^3^). Spontaneous locomotor activity was continuously monitored for 60 min using an automated video tracking system (Smart 3.0, Panlab Harvard Apparatus, Barcelona, Spain). The testing chamber was systematically sanitized with 75% ethanol between trials to eliminate olfactory interference. Animals demonstrating locomotor distances outside the 6000–8000 cm range on Day 3 were excluded from subsequent analyses. This pre-test acclimatization protocol ensured habituation to both injection procedures and experimental apparatus. Development phase (Day 4–8): Mice were randomly assigned to four groups: control, nicotine-treated, low-dose α-CTX TxIB (1 nmol/mouse), and high-dose α-CTX TxIB (5 nmol/mouse). The control group received a daily subcutaneous injection of saline at 10 mL/kg. The other groups were administered a single daily subcutaneous injection of nicotine (0.5 mg/kg). Immediately after injection, all experimental mice were transferred to the behavioral test box and allowed to move freely for 60 min. Withdrawal phase (Day 9–15): On Day 9, all experimental mice underwent a lateral ventricular catheterization and were allowed adequate recovery time.

Testing phase (Day 16): Thirty minutes prior to subcutaneous nicotine administration (0.5 mg/kg), mice received i.c.v. injections of either saline or TxIB (1 or 5 nmol/mouse). The control group received saline through both i.c.v. and s.c. routes. Spontaneous locomotor activity was quantitatively assessed for 60 min using the Smart 3.0 tracking system. Primary outcome measures included total distance traveled, zone transition frequency, and mean velocity.

### 4.4. Lateral Ventricle Cannula Implantation and Infusions

Lateral ventricular cannulation surgery and drug administration procedures were consistent with previous studies [27] in our laboratory. Briefly, after the mice were anesthetized, the scalp was clipped and the location of the tube placement was determined from the mouse brain atlas for the procedure.

During the testing phase, mice were anesthetized with isoflurane using the Mice and Rat Animal Anesthesia Machine (RWD, Shenzhen, China). Then, the drug was injected into the lateral ventricle via an automatic microinjection system (1 μL/min, 5 min).

### 4.5. Sample Preparation

After the testing phase, mice were euthanized by cervical dislocation, and their brains were excised. The VTA/NAc/PFC/CPU/HIP were dissected and aliquoted into microtubes according to the Allen Mouse Brain Common Coordinate Framework (CCFv362). Tissue samples were flash-frozen and stored at −80 °C for subsequent analysis.

### 4.6. BCA (Bicinchoninic Acid) Protein Assay

Samples were homogenized in pre-cooled PBS (5 μL/mg) containing 1% protease inhibitor (P1011, Beyotime, Shanghai, China) using a cryo-grinder with steel grinding beads (two 3 mm and one 4 mm; G0103-200G, G0104-200G, Servicebio, Wuhan, China). Following homogenization, the samples were centrifuged at 12,000× *g* for 10 min to remove the beads and cellular debris. The clarified supernatant was collected for subsequent analysis.

Protein concentrations were quantified using a BCA assay kit (C05-02001, Bioss, Shanghai, China) according to the manufacturer’s protocol. Briefly, 20 μL of each sample or standard was mixed with 200 μL of working solution in a 96-well plate and incubated at 37 °C for 30 min. The absorbance at 562 nm was measured using a SpectraMax M2 microplate reader (Molecular Devices, Sunnyvale, CA, USA) after equilibration to room temperature. Protein concentrations were calculated based on the standard curve.

### 4.7. Enzyme-Linked Immunosorbent Assay

Neurotransmitter concentrations (DA, NE, and 5-HT) were quantified using the enzyme-linked immunosorbent assay (ELISA) kit (XYM906261, XYM906271, XYM904431; X-Y Biotechnology, Shanghai, China) according to the manufacturer’s protocol. Briefly, 50 μL of standards or samples were incubated at 37 °C for 30 min, followed by five washes with PBS. Subsequently, 50 μL of HRP-conjugated specific antibodies were added and incubated under identical conditions. After additional washing steps, TMB substrate was added for color development. The absorbance at 450 nm was measured using a SpectraMax M2 microplate reader, and neurotransmitter concentrations were calculated using standard curves.

### 4.8. Western Blot

Protein samples were thawed on ice and mixed with 20% volume of 5 × loading buffer, followed by denaturation at 100 °C for 5–10 min. Equal protein quantities were separated by 10% SDS-PAGE (Mini-Protean, Bio-Rad, Hercules, CA, USA) and transferred to nitrocellulose membranes (Merck Millipore, Burlington, MA, USA) using a semi-dry transfer system (Mini Trans-Blot, Bio-Rad). Membranes were blocked with 5% skim milk in TBST (Tris-buffered saline with 0.1% Tween-20) for 30 min at room temperature, then incubated overnight at 4 °C with primary antibodies against NMDAR-2B (21920-1-AP, Proteintech, Wuhan, China), SERT (19559-1-AP, Proteintech), DAT (22524-1-AP, Proteintech), c-Fos (ABE457, Sigma-Aldrich, St. Louis, MO, USA), and GAPDH (10494-1-AP, Proteintech) diluted in TBST containing 3% BSA. After three TBST washes, membranes were incubated with HRP-conjugated goat anti-rabbit IgG (H + L) (SA00001-2, Proteintech) secondary antibody. Following additional washes, protein bands were visualized using enhanced chemiluminescence (AR1173, BOSTER, Wuhan, China) and imaged (Amersham Imager 600, Cytiva, Marlborough, MA, USA). Protein expression levels were quantified using ImageJ 1.54d software, normalized to GAPDH as the internal control.

### 4.9. Statistical Analysis

GraphPad Prism 6 (GraphPad Software, San Diego, CA, USA) was used for statistical analysis. Results are presented as mean ± standard error of the mean, and a *p*-value < 0.05 was considered statistically significant. One-way ANOVA was applied to compare means among groups.

## Figures and Tables

**Table 1:** Effect of TxIB on mouse behavior.

Behavioral Tests	Dose (i.c.v, nmol ^#^)	Results	Reference
Open field test (OFT)	1; 5	TxIB inhibited nicotine-induced locomotor sensitization	This study
Conditioned place preference (CPP)	0.1; 1	TxIB inhibited expression of nicotine-induced CPP	[27]
Conditioned place preference (CPP)	10	TxIB inhibited expression and acquisition of morphine-induced CPP	[26]
Morris water maze (MWM)	0.1; 1; 10	TxIB did not affect learning and memory in MWM	[26]
Elevated plus maze (EPM)	1	TxIB did not alter anxiety-like behavior in EPM	[26]
Open field test (OFT)	0.1; 1; 10	TxIB did not affect locomotor activity in OFT	[26]

# denotes nmol per mouse.

## Data Availability

The data presented in this study are available on request from the corresponding author.

## Abbreviations

The following abbreviations are used in this manuscript:

ACN	acetonitrile
BCA	bicinchoninic acid
BSA	bovine albumin
CPP	conditioned place preference
CPU	corpus striatum
CTx	conotoxin
Cys	cysteine
DA	dopamine
DAT	dopamine transporter
DβH	dopamine β-hydroxylase
DD	drug discrimination experiment
DDC	dopa decarboxylase
D1R	D1-dopamine receptors
D2R	D2-dopamine receptors
D3R	D3-dopamine receptors
D5R	D5-dopamine receptors
EDTA	ethylenediaminetetraacetic acid
EPM	elevated plus maze
GAPDH	glyceraldehyde-3-phosphate dehydrogenase
HIP	hippocampus
HPLC	high performance liquid chromatography
HRP	horse radish peroxidase
i.c.v.	intracerebroventricular injection
i.p.	intraperitoneal injection
MAO	monoamine oxidase
MWM	Morris water maze
NAc	nucleus accumbens
nAChRs	nicotine acetylcholine receptors
NE	norepinephrine
NIC	nicotine
NMDAR	N-methyl-D-aspartic acid receptor
OFT	open field test
PBS	Phosphate-buffered saline
PFC	prefrontal cortex
SA	self-administration
SERT	serotonin transporter
SPF	specific pathogen free
s.c.	subcutaneous injection
TBST	Tris buffered saline with Tween-20
TFA	trifluoroacetic acid
TH	tyrosine hydroxylase
Tris	tris(hydroxymethyl)aminomethane
VTA	ventral tegmental area
α-CTx	α-conotoxin
5-HT	serotonin
